# Structures of T7 bacteriophage portal and tail suggest a viral DNA retention and ejection mechanism

**DOI:** 10.1038/s41467-019-11705-9

**Published:** 2019-08-20

**Authors:** Ana Cuervo, Montserrat Fàbrega-Ferrer, Cristina Machón, José Javier Conesa, Francisco J. Fernández, Rosa Pérez-Luque, Mar Pérez-Ruiz, Joan Pous, M. Cristina Vega, José L. Carrascosa, Miquel Coll

**Affiliations:** 1 Centro Nacional de Biotecnología, (CNB-CSIC), Darwin 3, 28049 Madrid, Spain; 2 Institute for Research in Biomedicine (IRB Barcelona), The Barcelona Institute of Science and Technology, Baldiri Reixac 10, 08028 Barcelona, Spain; 3 Institut de Biologia Molecular de Barcelona (IBMB-CSIC), Baldiri Reixac 10, 08028 Barcelona, Spain; 4 Centro de Investigaciones Biológicas (CIB-CSIC), Ramiro de Maeztu 9, 28040 Madrid, Spain; 5 Present Address: Abvance Biotech srl, Ave. Reina Victoria 32, 28003 Madrid, Spain

**Keywords:** X-ray crystallography, Cryoelectron microscopy

## Abstract

Double-stranded DNA bacteriophages package their genome at high pressure inside a procapsid through the portal, an oligomeric ring protein located at a unique capsid vertex. Once the DNA has been packaged, the tail components assemble on the portal to render the mature infective virion. The tail tightly seals the ejection conduit until infection, when its interaction with the host membrane triggers the opening of the channel and the viral genome is delivered to the host cell. Using high-resolution cryo-electron microscopy and X-ray crystallography, here we describe various structures of the T7 bacteriophage portal and fiber-less tail complex, which suggest a possible mechanism for DNA retention and ejection: a portal closed conformation temporarily retains the genome before the tail is assembled, whereas an open portal is found in the tail. Moreover, a fold including a seven-bladed β-propeller domain is described for the nozzle tail protein.

## Abstract (web summary)

The genome of double-stranded DNA bacteriophages is delivered to host cells through the viral portal and tail structures. Here the authors describe structures of the bacteriophage T7 portal and the 1.5 MDa tail complex formed by the portal protein, adaptor protein and nozzle, providing insight into how the portal and tail machinery work during DNA packaging and ejection.

## Introduction

The order *Caudovirales* comprises the largest number of biological entities on Earth. They are bacterial viruses characterized by an icosahedral capsid, enclosing a double-stranded (ds) DNA, with a tail. These phages share a common assembly pathway of prohead formation and genome packaging with herpesviruses^1–3^. Mechanisms for DNA incorporation and ejection show a number of similarities based on the existence of a machinery built by several components, including the portal protein or head-to-tail connector, motor proteins that provide energy-dependent DNA translocation (terminases)^4^, and, in phages, the tail complex^5^. In the case of the *Podoviridae* family, bacteriophages have a short, non-contractile tail, which generally comprises an adaptor and a tubular nozzle or knob, with a plug to prevent DNA leakage. The other tail components are the fibers (or spikes), which are responsible mainly for bacterial receptor recognition^6,7^.

Phage portal proteins are key viral components located in a single pentameric vertex of the capsid and they act as initiators of capsid assembly. They are also critical components of the DNA-packaging complex and are involved in tail assembly^1,2,8^. In spite of a lack of extensive sequence similarity, all portal structures solved to date for *Caudovirales* (phi29^9,10^; SPP1^11^; P22^12^; T4^13^) share common morphological features, including a conical channel along the longitudinal axis and a conspicuous ring made of 12 subunits^1,2,8^.

DNA packaging into preformed proheads requires the interaction of the portal protein with the terminase, which generates forces involved in the processive translocation of dsDNA into the viral capsid, where it is stored at quasi-crystalline concentration^1,14,15^. Both in phages^16–18^ and in herpesviruses^19^, the interaction of the packaging terminase occurs at a region of the portal protein that extends outside the capsid shell through the portal vertex. After completion of the packaging, the DNA stored inside the capsid undergoes considerable stress due to mechanical strain induced by bending, as well as extensive repulsive electrostatic interactions^20–22^. In phi29 and SPP1, the DNA interacts with positively charged residues in the central channel of the portal protein, which have been proposed to contribute to stabilize the DNA inside the capsid prior to tail assembly^9,22,23^.

The DNA is permanently stabilized inside the capsid, after the release of the terminase, by the subsequent incorporation of a dodecameric adaptor complex and the rest of the tail machine^24,25^. In phage P22, the gp4 adaptor ring interacts at the outer tip of the portal protein, called the clip, and has a long C-terminal helix that extends onto the outer surface of the portal protein monomer–monomer interface^12^. Although the structure of the isolated adaptor protein of phage Sf6 (gp7) shows a very similar arrangement to that of P22 gp4 and other adaptor proteins^12,26^, it shows differences in the relative position of the first α-helix. These observations suggest that this conformational change might be related to the structure before and after assembly in the mature phage^26^.

In phage T7, the formation of the tail starts by the assembly of the gp11 adaptor toroidal ring, after which protein gp12 assembles on the distal side of the adaptor to build the hexameric nozzle^27^. The interaction of the adaptor and the nozzle generates the six regions where the fibers (trimers of gp17) assemble to render the final functional viral particle^27,28^. *Podoviridae* nozzles present distinct conformations during DNA ejection. In T7, interaction of the fibers with the bacterial receptor triggers a conformational change by untwisting the nozzle monomers, which results in the opening of the channel required for DNA release^28,29^. Although the precise details and molecular mechanisms of DNA release are not fully known, similar conformational changes in the nozzle have also been characterized in phage P-SSP7, a relative of T7^30^. In P22, the tail machinery also undergoes conformational changes during bacterial adsorption^31^.

Here we report a number of crystal and high-resolution cryo-electron microscopy (cryo-EM) structures of the T7 bacteriophage portal (gp8). The structures show two conformations—open and closed—of the portal and suggest a possible mechanism of the channel valve that regulates DNA passage. Moreover, we describe the atomic structure, determined by cryo-EM, of the 1.5 MDa T7 tail complex (gp8-gp11-gp12), thus characterizing the whole ejection channel. In particular, the tail nozzle (gp12) shows an unexpected fold with six β-propellers, which are essential to tightly close the channel gate in the mature phage. All these structures, associated with different states of the infection cycle, support a mechanism underlying DNA retention inside the capsid and its ejection during infection.

## Results

### Structure of the T7 bacteriophage portal

Several X-ray data sets from various crystal forms of the T7 portal in its dodecameric (12mer) and tridecameric (13mer) forms were collected. Although the portal protein is always found as a dodecamer in virions, assemblies containing 11–13 subunits have been described for other portals when expressed under non-physiological conditions^13,32^. Despite extensive efforts, all attempts to determine the phases of any of the data using heavy-atom methods failed. The T7 portal structure was finally solved by combining cryo-EM and X-ray crystallography. An initial map of the tridecameric form of the gp8 protein at 5.8 Å resolution was obtained by cryo-EM (gp8-13mer_EM_; Supplementary Table 1 and Supplementary Fig. 1). The ring-shaped volume allowed the ab-initio building of a partial poly-alanine model composed mainly of α-helices, which contained 36% of the structure. This partial model was later used for phasing a 3.4 Å resolution gp8-13mer crystallographic data set by molecular replacement and a full atomic model was built into the resulting electron density map (gp8-13mer_cryst_; Supplementary Table 2 and Supplementary Fig. 2). The gp8 monomeric structure was then used for phasing the gp8-12mer 3.6 Å resolution crystallographic data (gp8_closed_, see Methods section), which yielded an atomic model of the dodecameric portal (Supplementary Table 2).

**Fig. 1:**
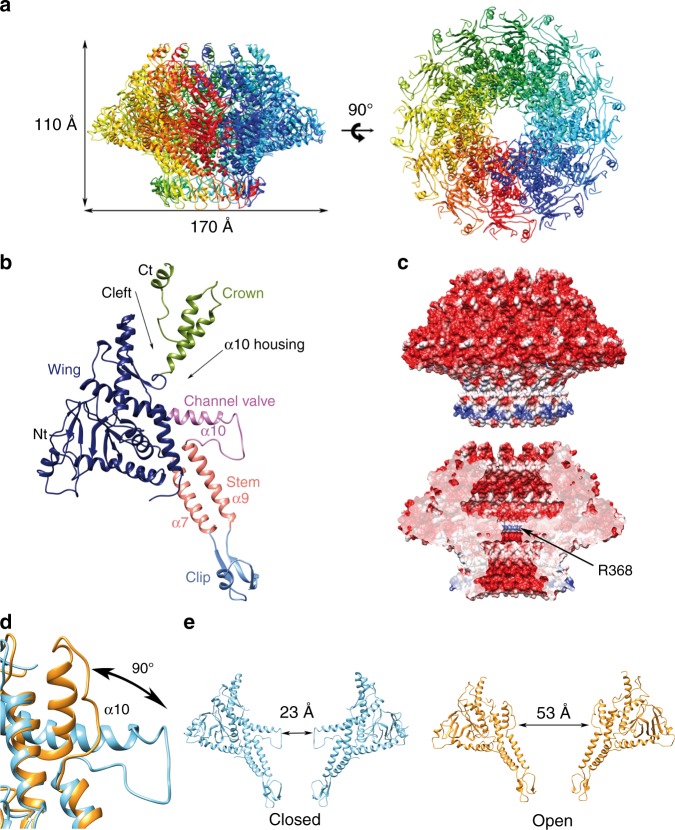
Structure of the T7 bacteriophage portal. **a** Ribbon representation of the gp8_closed_ structure of the portal with rainbow coloring by monomer. The dimensions of the particle are indicated. Left, lateral view; right, axial view. **b** Ribbon representation of a monomer of the gp8_closed_ structure, colored by domains, with relevant secondary structure elements and structural features indicated. **c** Electrostatic potential on the external (up) and inner channel (down) surfaces of the gp8_closed_ portal. A thin positively charged channel ring, formed by R368, is indicated. **d** Detail of the superposition of gp8_closed_ (blue ribbon) and gp8_open_ structures (orange ribbon). The conformational change of the channel valve is indicated with an arrow and maximum torsion angle is shown. **e** Comparison of the two conformations by showing two opposed monomers. Left, closed conformation shown in blue ribbon. Right, open conformation shown in orange ribbon. The crown domain is not visible for the open conformation and has not been shown for the closed connector for the sake of clarity

The overall shape of the T7 portal protein shows a ring-like assembly formed by 12 subunits and with an axial central channel. The external diameter of the particle is 170 Å, whereas its height is 110 Å. The diameter of the channel (measured between opposite Cα atoms) is 23 Å at its narrowest section, which would hinder the passage of a B-DNA molecule (Fig. 1).

The structure contains four domains equivalent to those found in other viral portals: the stem, the clip, the wing, and the crown (Fig. 1b). The wing, which has a unique conical shape not found in any of the previously described bacteriophage portals, is the largest domain and it protrudes outwards at the middle section of the assembly. It contains the N-terminal end of the protein, which is located at the outer surface. The wing is built of six α-helices, three of them forming an up–down α-bundle, and a β-sandwich formed by two perpendicular β-sheets of seven and three anti-parallel β-strands, respectively. This fold is reminiscent of the SH3 domain, as already described for the phi29 portal^9^. In addition, there is a two-stranded anti-parallel β-sheet at the wing/crown cleft. The clip, which is found at the “bottom” of the particle and points toward the exterior of the viral capsid, contains three β-strands, which perform intra- and inter-subunit interactions, and a short α-helix. The stem connects the wing and the clip, each monomer comprising two tilted α-helices (α7 and α9) in an anti-parallel disposition, forming a double-layer 24-helix ring around the channel, in the particle. A 39-residue helix, α10, perpendicular to the channel axis, connects the channel with the outer part of the wing domain. The tunnel loop, a singular feature also found in other portal particles, is between α9 and α10, and only partially observable in the gp8 dodecameric structure due to its intrinsic flexibility. Finally, the C-terminal part of the protein forms the crown domain, which in the mature virus is located inside the capsid shell and interacts with the core proteins. A deep cleft separates the wing and crown domains, which are connected only by a hinge at G434 between strand β13 of the wing and helix α11 of the crown. This feature confers some freedom of movement to the crown domain around that point, which is also supported by the fact that monomers from the dodecameric and tridecameric structures are very similar, except for the relative position of the crown domain respect to the rest of the protein (root mean squared deviation (RMSD) 0.849 Å). The crown was predicted to contain long helical structures, resembling the barrel domain described for other viral connectors^12^, but it was found to be partially disordered in all our structures, with the last 42 residues not visible in the density maps. The upper part of the crown may become ordered only upon interaction with the core viral proteins. Regarding the shape of the portal central channel, there are two cavities, “upper” and “bottom”, with a conical and an inverted conical shape, respectively, separated by the protrusion of α10 toward the center of the channel. The electrostatic potential of the protein is markedly negative, both on the external and inner surfaces, except for the exterior of the clip and the tunnel loop, due to R368 (Fig. 1c).

### α10 acts as a valve, opening and closing the channel

A second cryo-EM data set yielded a higher resolution map than the initial one used for phasing. This map at 4.1 Å resolution shows a different conformation of the 12mer portal (gp8_open_; see below, Supplementary Table 1 and Supplementary Fig. 3). The overall structure is similar to that already described (RMSD 1.03 Å), with the exception of the conformation of the channel α10 helix, which is kinked in the middle and deviates “upwards,” instead of pointing perpendicular toward the channel axis (Fig. 1d). The kink occurs at a region containing two adjacent glycine residues (G386 and G387), which provide the necessary flexibility to change the orientation of the N-terminal half of the helix. The movement represents a large swing of 90°. The density corresponding to the tunnel loop is partially visible and reveals that it is in an extended conformation, allowing the connection of the now more distant α9 and α10 helices. Although it was previously hypothesized that a kink on the tunnel loop helix may be related to its ability to adopt distinct conformations during DNA packaging and retention^11^, here we observe experimentally such notable conformational change.

This conformational change results in a substantial increase in the channel diameter, from 23 Å (α10 extended) to 53 Å (α10 kinked). Therefore, two clearly different conformations in the T7 connector were defined: one closed (gp8_closed_) and the other open (gp8_open_) (Fig. 1e). In gp8_open_, the kinked part of helix α10 occupies the “upper” cavity of the channel or α10 housing cavity (Fig. 1b), thus defining a larger conical channel instead of the two inverted conical cavities observed in gp8_closed_. Although the first structure described in this work (gp8_closed_) would impede the DNA passage through it, the second structure (gp8_open_) would allow it. The existence of two well-defined portal conformations allows this protein to act as a valve at the portal pore, regulating the passage of DNA into the capsid.

### Structure of the T7 tail machine

**Fig. 2:**
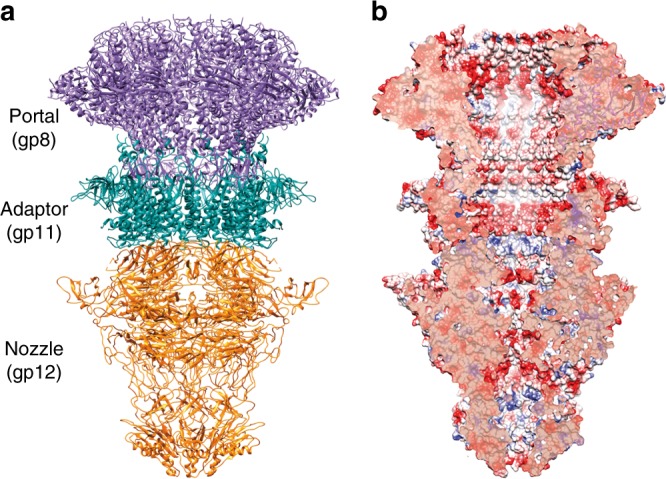
Structure of the T7 tail. **a** Ribbon representation of the cryo-EM T7 tail structure (gp8-gp11-gp12). Gp8 portal, gp11 adaptor, and gp12 nozzle proteins are shown in purple, green, and orange, respectively. **b** Longitudinal cut of the electrostatic potential surface

The T7 tail machine is composed of four proteins^27^: the portal (gp8), the adaptor (gp11), the nozzle (gp12), and the fibers (gp17). We solved the structure of the fiber-less tail (1.5 MDa) by cryo-EM at 3.3 Å resolution (gp8-gp11-gp12; Fig. 2a, Supplementary Table 1, and Supplementary Fig. 4). This complex shows a tubular conical shape 293 Å long and 175 Å wide, organized in two 12-fold rings (gp8 and gp11) and a 6-fold nozzle (gp12). The structure presents two invaginations on the external surface. The first, placed between the portal and the adaptor, serves for capsid docking, whereas the second, between the adaptor and the nozzle, is the interaction surface of the fibers (Fig. 2).

The central channel of the tail is closed at the hexameric nozzle at different gates that retain the DNA inside the capsid in the mature phage. This channel is mainly negatively charged, a feature that has been proposed to be essential to avoid DNA sticking during ejection^8^ (Fig. 2b).

**Fig. 3:**
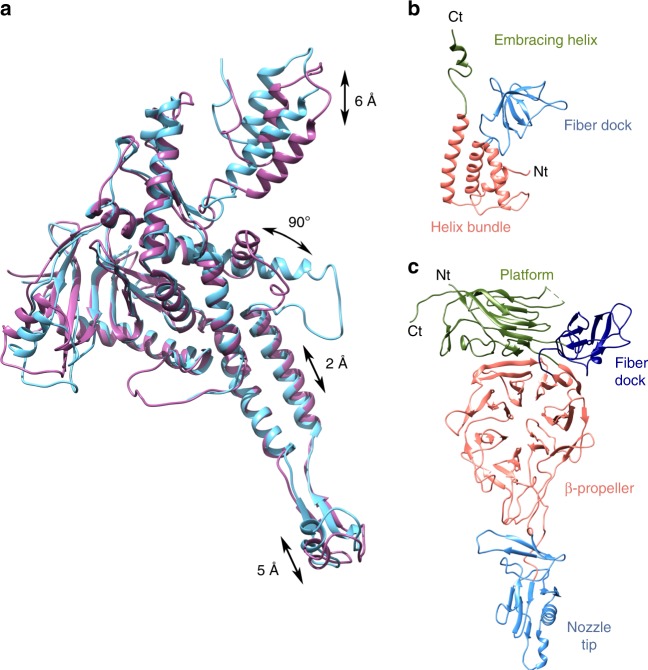
Atomic structures of T7 tail proteins. **a** Superposition of the gp8_open_ as in the tail complex (orchid purple) and gp8_closed_ (blue) portal monomers. Distances and angles of the moving regions are indicated. **b** Ribbon representation of gp11 adaptor protein monomer. The different domains are colored and labeled. **c** Ribbon representation of gp12 nozzle protein monomer. The different domains are colored and labeled

The portal protein present in the tail was traced using the gp8-free protein as template, which was solved previously (see above). The channel valve of the portal was found in its open conformation, which should allow the free passage of DNA into the ejection channel. Superimposition of the gp8_closed_ structure found in the free portal with the gp8_open_ portal present in the tail complex revealed, in addition to the channel valve movement, additional movements affecting various substructures of the protein (Fig. 3a, Supplementary Video 1, and Supplementary Video 2). The largest movement occurs at the crown (6 Å) and at the hinge within the wing/crown cleft, correlated with the opening/closing of the portal valve, as the helix valve α10 in the gp8_open_ conformation would clash with the hinge in gp8_closed_ conformation. There is also movement of the clip (5 Å), caused by the interaction with the gp11 adaptor in the assembled tail. This movement seems to be transferred “upwards” through the stem helix α9 (2 Å), possibly pushing the valve up to its open state.

The gp11 adaptor protein is assembled to the portal clip region, forming a 12-fold conical ring. The structure of the monomer is composed of five α-helices and five β-strands, divided into three domains as follows (Fig. 3b): (i) an α-helix bundle (α1–α4), which creates a wide central channel in the dodecamer with a marked electronegative surface 40 Å in diameter in its narrowest section, and no constraints (Fig. 2b); (ii) a fiber dock, which constitutes one of the fiber interaction regions (see below for a second fiber dock in the tail nozzle); and (iii) a C-terminal embracing helix, which surrounds the portal protein stem. Despite the lack of sequence homology, all adaptor proteins described to date present the same organization of four α-helices in an up–down bundle (Supplementary Fig. 5)^12,26,33–35^. In the case of T7, the α-helix bundle is stabilized by a disulfide bond between α3 and α4 (C133–C171). The fiber dock domain is less common and is not present in HK97, SPP1, P22, or Sf6 bacteriophages, probably due to the different morphology of the tail and fiber interactions in these viruses. The fiber dock has a triangular shape and is formed by five anti-parallel β-strands disposed in a small jelly-roll β-barrel. The C-terminal α5 that forms the embracing helix points toward the portal protein; this helix is replaced by a flexible stretch in other bacteriophages (Supplementary Fig. 5)^26^. The ring assembly relies mainly on electrostatic interactions between monomers that have a bipolar surface, with one side mainly electronegative and the other electropositive (Supplementary Fig. 5)^26^.

The gp12 nozzle protein forms a hexamer attached to the bottom of the adaptor protein. Each monomer is composed of 62 β-strands and 2 α-helices, one of them forming part of the nozzle tip together with a 3_10_ helix (Fig. 3c). The gp12 fold shows no structural similarity with any bacteriophage tail protein previously reported. It is organized around a large central β-propeller domain. Three other domains are also present: (i) the platform, which interacts with the adaptor protein; (ii) the fiber dock, which is the fiber interaction domain (the fiber is fitted in between the nozzle fiber dock and the adaptor fiber dock); and (iii) the nozzle tip domain at the most distal part (Fig. 3c).

The central β-propeller domain has a diameter of ~40 Å, arranged into seven blades, each with the characteristic four anti-parallel β-strands (Fig. 3c and Supplementary Fig. 6). The propeller is open at two points. The “upper” opening point is stabilized by the so-called velcro closure in blade 1^36^, where the last β-strand of the blade is actually the first strand in the sequence of the propeller, thus gluing the N- and C-terminus of the domain. The propeller is further stabilized in this region by a disulfide bond between residues C504 and C554, which belong to blades 7 and 1, respectively (Supplementary Fig. 6). The “bottom” opening is in fact a loop of blade 3 that extends out and leads to the nozzle tip domain. It is stabilized, among other interactions, by hydrophobic interactions at the base of the propeller (Y146, Y281, and W297) (Supplementary Fig. 6). Although there is no significant sequence homology, the P22 tail protein gp10 shows by structure prediction the possible existence of a β-propeller domain (I-TASSER, data no shown). As this protein is one of the most conserved genes in all Podoviridae^37^, it is possible that β-propeller domains might play a common role in DNA ejection. The nozzle tip domain is a β-barrel (with an α-helix instead of one of the barrel β-strands), followed by a two-turn 3_10_ helix, which is the very tip of the tail and thus protrudes toward the bacterial outer membrane during infection. A similarity fold search of this domain using Dali^38^ retrieved, among others, the gp11 protein of T4 bacteriophage base plate, even though they do not have any sequence similarity. T4-gp11 is essential for DNA ejection and plays a key role during fiber attachment^39^. In addition to the nozzle tip β-barrel, there is a three-stranded β-sheet that acts as an intervening structure between the nozzle tip and the β-propeller.

The fiber dock connects the end of the β-propeller to the platform domain and is situated at the outer surface of the assembly (Fig. 3c). This domain is triangular and is composed of a small α-helix and six anti-parallel β-sheets, forming a jelly-roll β-barrel. Finally, the C-terminal domain forms the platform, which includes nine long anti-parallel β-sheets forming another, larger, jelly-roll β-barrel. Of note, three of the strands of this barrel belong to the 45-residue N-terminus of the protein. This observation suggests a possible circular permutation event where the C-terminus of the gene has moved to the beginning of the sequence.

In the hexameric nozzle structure, it is apparent that the elongated protomers are highly twisted. The twist is left handed and about 45° when comparing proximal and distal sections of the nozzle.

### Characterization of tail portal and adaptor interaction

**Fig. 4:**
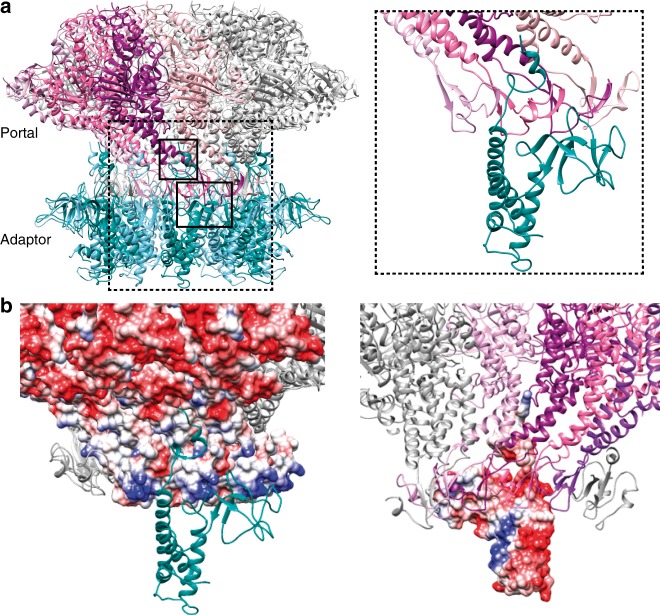
Tail portal-adaptor interaction. **a** Ribbon representation of the gp8 portal and gp11 adaptor proteins in the tail complex. Left, binary gp8-gp11 complex; right, close-up of the interaction region. In order to facilitate the interpretation of the image, only one subunit of the gp11 oligomer is shown in the close-up view. The adaptor subunits are shown in dark green and light blue; the four subunits of gp8 interacting with gp11 subunit highlighted in the right panel are shown in plum, magenta, purple, and pink, whereas the rest of the subunits are indicated in grey. **b** Surface charge distribution of the portal/adaptor interaction. In order to facilitate the interpretation, a single monomer of gp11 protein is shown. Left, view from the outside the complex showing the portal electrostatic surface and a ribbon representation of gp11. Right, view from the inside of the channel, showing the adaptor electrostatic surface, and a ribbon representation of the portal protomers

The presence of the portal ring is necessary to build the gp11 dodecameric ring, otherwise gp11 behaves as a monomer^27^. Gp11 interacts at the “bottom” of the gp8 portal by strangling its clip domain between the embracing helix, the C-terminus of the preceding helix (which forms the internal channel surface of the adaptor), and the fiber dock, knitting an extensive network of electrostatic interactions and hydrogen bonding. In fact, the clip domains fit inside the inner channel of the adaptor, at its wider “upper” side. As the monomers are inclined in different directions in gp8 and gp11, a single gp11 monomer interacts with four gp8 monomers (Fig. 4a), as also described for bacteriophage P22^12^. A gp11 monomer presents a higher surface of interaction with gp8 (1771 Å^2^) than with its adjacent gp11 monomer (1435 Å^2^). This observation, together with the poor hydrophobic inter-monomer contacts in gp11, would explain why this protein readily forms an oligomeric ring on gp8 but is a monomer in its absence. There are two main interaction regions between gp8 and gp11 (Fig. 4a): one at the clip of the portal, where residues in the gp11 fiber dock (E173, E175, and D177) interact with the gp8-positive belt in the clip domain (Q307, R309, R310, and K313) (Fig. 4b), and the other one at the portal stem, where the gp11 embracing helix residue (R196) reaches portal α7 (residues D275 and D278) (Fig. 4b). A similar interaction at the portal “bottom” was described for the T4 portal-terminase complex^13^, suggesting that both the terminase and the adaptor proteins use a similar docking mode onto the portal. The second interaction point might be accessible only after full DNA packaging into the capsid, when DNA pressure extrudes the T7 portal from the capsid^40^ (see below).

### gp12-nozzle assembly and its role in securing DNA

**Fig. 5:**
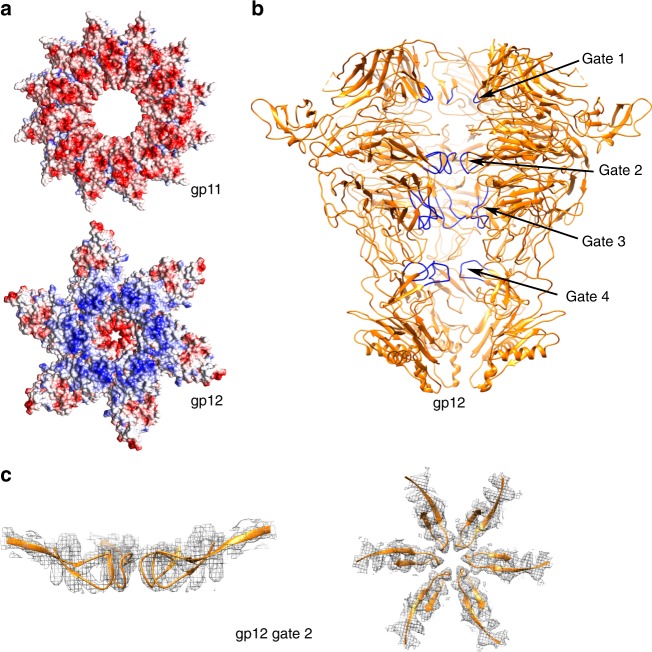
Structural characterization of the nozzle protein. **a** Electrostatic potential surfaces of the interacting region between the gp11 adaptor (top) and gp12 nozzle (bottom). **b** Ribbon representation of gp12 protein structure in the tail complex in orange, with the four closing gates highlighted in dark blue. Diameters of the channel at each of the gates when measured from Cα to Cα are as follows: gate 1, 18.3 Å; gate 2, 8.6 Å (from carbonyl O to carbonyl O); gate 3, 13.8 Å; and gate 4, 23 Å. **c** Detail of the gp12 closing gate 2, with the map density shown in mesh and the protein atomic model in orange ribbon. Left, lateral view; Right, axial view

The nozzle protein is assembled as a tubular hexamer at the virus tail, lowering the 12-fold symmetry of the portal and the adaptor to the 6-fold symmetry characteristic of the tail machinery^27^. Gp11 adaptor and gp12 nozzle present a large network (1252 Å^2^) of electrostatic interactions between them. Although the “bottom” part of the adaptor is mainly electronegative, the “upper” part of gp12 is highly electropositive (Fig. 5a). In order to switch the symmetry from 12 to 6, a gp12 platform domain has to interact with two gp11 monomers (Supplementary Fig. 6b). The two gp11 monomers engage for the interaction of acidic residues from the loop between helices α1 and α2 (E26, D35, D39), which is found in two alternating conformations laying on distinct regions of the gp12 platform; thus, each of the two contiguous gp11 monomers interacts with a different set of positive residues of gp12 (Supplementary Fig. 6b).

The oligomeric assembly strategy of gp12 differs greatly to that of gp11 and gp8, the latter two showing a bipolar distribution of the electrostatic charges in either side of the protomer. In contrast, gp12 charge distribution is more heterogeneous and the inter-subunit interactions are held mainly at three distal points, namely the platform, β-propeller, and nozzle, leaving the rest of the structure relatively loose and free to potential movements. The strongest interaction point between monomers is located at the β-propeller domain, through the protruding loops between the blades. The β-propellers are placed with their planes radial-wise, defining a section of the particle that resembles a six-pointed star (Fig. 5a).

In the mature virus, the gp12 nozzle protein is the main molecule responsible for closing and securing the DNA inside the tail. This task is mediated by four closing gates with negatively charged residues found along the gp12-internal channel (Fig. 5b). The first DNA barrier is located at the platform level, where the aperture is 18.3 Å. It is followed by the most constricted part of the channel, at the β-propeller level, with two narrowings of 8.6 Å and 13.8 Å, respectively. The 8.6 Å aperture gate is remarkable, as it is formed by the main chain carbonyl group of D478, at the tip of a loop of blade 6 of the β-propeller (Fig. 5c). This is a tight loop without any flexibility, well stabilized by several hydrophobic residues, where there is no room for local movement to increase the aperture. The opening of this gate must involve the displacement of the large β-propeller domains or entire protomers. Finally, there is one further narrowing (gate 4) at the nozzle tip with an aperture of 23 Å.

## Discussion

A number of reports describe several conformations of the portal protein that might be related to its function. The portal complex of P22 is found in two conformations in proheads and mature heads, with different affinities for other viral components, such as the capsid, scaffold, or terminase^24,41^. In the case of phage SPP1, rearrangements of the portal protein subunits are essential for DNA translocation^42^ and they are related to the interaction with DNA in the inner channel of the portal assembly^23^. These changes, leading to a reorganization of the portal channel, suggest that, rather than being a passive DNA pore, the portal has an active role during packaging, serving as a sensor device in the determination of the amount of DNA to be packaged^23,42,43^. The importance of subtle conformational changes in the context of DNA translocation has also been claimed in modeling studies. In this regard, the distribution of regions differing in the degree of stiffness, thus allowing specific compressions and DNA-dependent distortions, as well as the existence of quasi-equivalent contacts, have been proposed to play important roles in DNA packaging^43–45^. Recently, it was also described for bacteriophage P23–45 that the portal may adopt different conformations depending on the stage of virus assembly, with synchronized movements of different domains communicating the inner part of the capsid to the exterior^46^. In this study, we have described two distinct conformations, namely open and closed, of the T7 bacteriophage portal. Docking of the closed conformation found in portal crystals into proheads from two different viral systems (T7 and P23–45) shows that this conformation is compatible with the portal region of the viral proheads before DNA packaging (Supplementary Fig. 7). Comparison of the two portal conformations has highlighted the α10 helix-tunnel loop as a region of the protein that is likely to act as a channel valve during viral morphogenesis. The closure of the T7 portal protein, as observed here, does not allow the passage of the genome and therefore the portal valve is likely to be in the open conformation during DNA packaging. Although we cannot rule out the loss of rotational symmetry of the particle during DNA translocation or that some of the monomers differ to others in their α10 helix-tunnel loop conformation, the present structures do not provide any indication for distinct conformations in any given particle: the valve is either fully open or fully closed. Thus, the hypothesis of an undulating belt tightly embracing the DNA^11^ as it runs along the channel cannot be inferred from the present study.

**Fig. 6:**
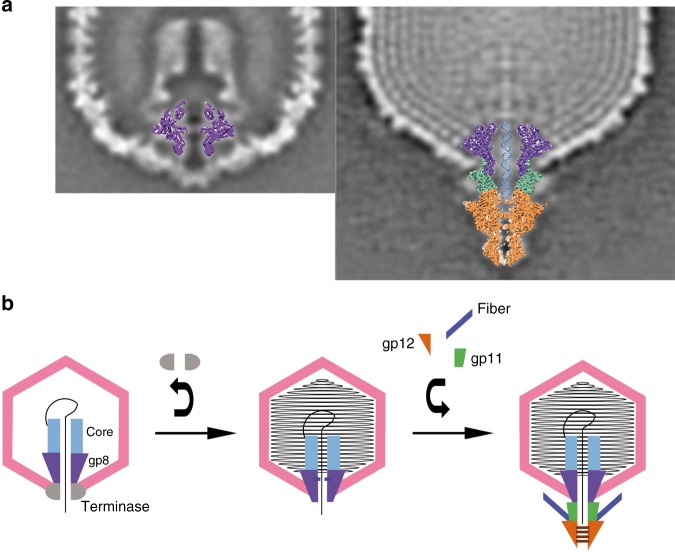
Proposed model for T7 bacteriophage DNA securing inside the capsid. **a** Overlapping of the “free” portal into the prohead (left) and the tail complex into the mature virus (right) from central sections through the reconstructions described in ref. ^40^. **b** Scheme showing the T7 bacteriophage assembly pathway. The capsid is shown in pink, the portal (gp8) in purple, the core complex in light blue, the terminase in gray, the adaptor (gp11) in green, the nozzle (gp12) in orange, the fibers in dark blue, and the DNA in black. The portal channel valve can be either open or closed. When the portal is in the prohead, the terminase–portal interaction stabilizes the open conformation, allowing DNA packaging. When the terminase leaves the complex, the portal channel valve closes, thus preventing DNA leakage from the capsid. The interaction of the portal with the adaptor protein re-establishes the open conformation of the portal channel valve, permitting the DNA to slip along the tail channel up to the nozzle, ready for ejection. In the mature virus, the gates of the nozzle protein close, retaining the DNA in the tail channel. These gates are closed until the reorganization of the nozzle (untwisting), which is triggered by the interaction of the tip and the fibers with the host membrane. The gates then open and the viral genome is ejected

Once DNA translocation ends, the portal is extruded from the capsid, thus exposing the clip and stem domains (Fig. 6a)^40^. This exposure in turn allows the portal to interact with the adaptor gp11, replacing the terminase. The accessibility of the newly exposed surfaces, together with the symmetry matching (C12) between the adaptor and the portal, might favor this interaction over the terminase. The terminase detachment signal may be caused by a conformational transition of the portal, mediated by a sensing signal induced by the DNA pressure^24^, thus allowing portal interaction with the adaptor and the assembly of the rest of the tail components^1,25^. The flexible crown domain, whose movement is observed in our structures, may be involved in the process of sensing DNA pressure^24,47^. The movement of the crown would then be transmitted to the channel valve, closing it. Both movements, crown and valve, are correlated in our structures. Thus, during the brief time where no protein is docked to the portal, the valve is closed, thereby preventing leakage of the packaged DNA. Temporary retention of the DNA inside the capsid in the absence of other proteins was reported for phi29 bacteriophage^22^, an observation that supports our hypothesis and the role of the channel valve. In the case of Epstein–Barr herpesvirus, the recently determined portal structure^48^ shows an equivalent diaphragm-like structure in the channel that could play a similar valve role for DNA retention during packaging. An open question is whether the valve closes onto the DNA, which may occupy the whole portal channel up to the clip when the translocation finishes. The valve would thus trap the DNA by the interaction of the arginine side chains (R368) of the tunnel loop with the DNA phosphates. The observation that the closed conformation of the channel valve leaves an aperture that is very similar to the diameter of the DNA suggests this hypothesis.

Although the free portal can be found in the two conformations (open and closed), the interaction with the adaptor protein clearly stabilizes the open conformation (probably through the clip-α9-α10 path), allowing the DNA momentarily retained inside the portal to slip further through the channel, pass the wide adaptor section, and reach the nozzle. In the mature virus, the genome is retained inside the ejection channel and stopped from leaking at the nozzle level by the four gates. As the channel is tightly blocked, in particular at the stiff gate 2 made by the β-propellers, which shows minimal aperture, the genome is fully secured inside the capsid, and thus a stable infective virus is ready. Closing of the DNA ejection channel by β-propeller loops might be also present in other phages. In the case of P22, there is a highly conserved hexameric tail protein (gp10)^37^, which connects the adaptor and the tail needle^31^, which also shows propeller-like domains by structure prediction studies.

When a new host is reached, the interaction of the tail fibers with the bacterial outer membrane receptor triggers a conformational change in the nozzle, which results in the opening of the channel required for DNA release^28,29^. Our structure of the tail shows that gate 2 cannot be opened by a local (i.e., loop) movement. A large displacement of the protomers is necessary, involving the separation of the six β-propeller domains and the consequent expansion of the channel. In a previous low-resolution cryo-EM study^29^, we observed this large conformational movement, which involves the untwisting of the six elongated protomers. The present high-resolution structure further supports this observation.

All together, our results allow us to propose a model for the roles of each tail protein during T7 bacteriophage DNA packaging, retention, and ejection (Fig. 6b). From a methodological point of view, this work illustrates the power of the correlative combination of X-ray crystallography and cryo-EM techniques, in order to solve challenging molecular structures.

## Methods

### Protein expression and purification

The T7 bacteriophage *gp8* gene was inserted into the pET28a vector (Novagen), between the NcoI and NotI restriction sites. The protein was expressed in *Escherichia coli* BL21(DE3) grown at 37 °C, after induction with 0.4 mM of isopropyl β-d-1-thiogalactopyranoside (IPTG) at an optical density (OD) of ~0.6, for 3 h at 37 °C or overnight at 16 °C. Cells were resuspended in 20 mM Tris-HCl pH 8.0, 500 mM NaCl, 3 mM β-mercaptoethanol, 20 mM imidazole, 40 µg/ml DNase I, and Complete Protease Inhibitor Cocktail Tablets (Roche), and lysis was performed using a cell disruptor (Constant Systems, Ltd.). The sample was then clarified after centrifugation for 30 min at 30,000 × *g* and purified by a three-step protocol^13^. Briefly, the protein was loaded onto a HisTrap HP column equilibrated in buffer A (20 mM Tris-HCl pH 8.0, 500 mM NaCl, 3 mM β-mercaptoethanol, 20 mM imidazole) and eluted in buffer A with 350 mM imidazole; the protein was then loaded onto a Sephacryl S-400 column, followed by a Superose 6 column, equilibrated in buffer B (20 mM Tris-HCl pH 8.0, 500 mM NaCl, 5 mM dithiothreitol, 2 mM ethylenediaminetetraacetic acid). The sample was concentrated with a 30,000 Da molecular weight cutoff Vivaspin (GE Healthcare). In some cases, the protein was dialyzed in TMS buffer (50 mM Tris-HCl pH 7.8, 100 mM NaCl, 10 mM MgCl_2_) before grid preparation.

*gp8*, *gp11*, and *gp12* genes were cloned in tandem in the p-RSETB vector^27^. The tail complex was expressed in *E. coli* C43 after induction with 1 mM IPTG for 3 h at OD_600_~0.4. Cells were resuspended in TMS buffer with Complete Protease Inhibitor Cocktail Tablets (Roche) and sonicated. The complex was purified in two steps: proteins were first loaded onto a HisTrap HP and eluted in TMS buffer with 200 mM imidazole, and then onto a Superose 6 column in TMS. The complex was concentrated using a 50 k Amicon Ultra (Millipore).

### Cryo-EM sample preparation and imaging

R2/2 Quantifoil grids were glow-discharged for 1 min for the gp8 protein, while they were cleaned with acetone and treated with 0.1% w/v poly-l-lysine (Sigma) for 1 min in water for the tail complex. Next, 3 µl of purified sample (at 3 mg/ml or 1.5 mg/ml for gp8, and 0.8 mg/ml for the tail complex) was pipetted onto the grids and incubated for 3 min at 22 °C, at 95% humidity. Grids were then blotted for 3.5 s, with forces between −3 and −5 on a FEI Vitrobot Mark IV (FEI), and plunge-frozen in liquid ethane.

A gp8 grid at 3 mg/ml was transferred to a FEI Talos Arctica (FEI) electron microscope operated at 200 kV at the Cryo-EM Centro Nacional de Biotecnología-Centro de Investigaciones Biológicas CSIC facility in Madrid (Spain). A total of 1065 movies fractioned in 26 frames were recorded in an automated fashion on a Falcon II (FEI) detector, using EPU (FEI) with a pixel size of 1.37 Å/pix, fraction exposure time of ~0.058 s/frame, and a total accumulated dose of ~22.8 e^−^/Å^2^(~0.88 e^−^/Å^2^/frame). Gp8 grids at 1.5 mg/ml in TMS buffer were transferred to a FEI Titan Krios (FEI) electron microscope operated at 300 kV at the European Molecular Biology Laboratory in Heidelberg (Germany). Images were recorded in an automated fashion on a Gatan K2 Summit (Gatan) detector, with a pixel size of 1.04 Å/pix using SerialEM^49^. A total of 4517 movies were collected fractioned in 40 frames, with a fraction exposure time of 0.5 s and a total accumulated dose of ~39.4 e^−^/Å^2^ (0.985 e^−^/Å^2^/frame). Tail complex grids were transferred to a FEI Titan Krios (FEI) electron microscope operated at 300 kV at the Electron Bio-Imaging Centre (eBIC), Diamond Light Source in Didcot (UK). Images were recorded in an automated fashion on a Gatan K2 Summit (Gatan) detector, with a pixel size of 1.048 Å/pix using EPU (FEI). A total of 2744 movies were collected fractioned in 40 frames, with a fraction exposure time of 0.2 s and a total accumulated dose of ~33.6 e^−^/Å^2^(0.84 e^−^/Å^2^ /frame).

### Image processing and map calculation

Cryo-EM data processing was performed using the Scipion software framework^50^. Dose-fractionated image stacks were motion corrected and dose-weighted using MotionCor2^51^. Defocus was estimated using ctffind4 and xmipp3^52,53^ for the gp8 Talos Arctica data set, and GCTF program^54^ for the gp8 and tail complex Titan Krios data sets, and Contrast transfer function (CTF) was corrected in the reconstruction process by RELION. Particles were picked using xmipp3^53^ for gp8 and gautomatch for the tail complex. Extracted particles were classified using RELION 2D and 3D^55,56^, and initial volumes were built using Ransac^53^. In the case of the gp8 Titan data, 2D average visual inspection was used to select classes corresponding to frontal and partial frontal views of the dodecameric particles and to discard those corresponding to tridecamers. The particles used in the final reconstruction were selected after 3D classification of the selected frontal and partial frontal views plus the lateral views. Although C12 or C13 symmetries were applied for gp8 reconstruction, C6 symmetry was applied for the gp8gp11gp12 complex. Final volumes were obtained using RELION Auto-refine with 12,642 and 32,388 particles for gp8 Talos Arctica and Titan Krios acquisition data sets, respectively, and with 92,382 particles for the tail complex. RELION Post-processing was used for the gp8 Talos Arctica data set. In all the cases, structure resolutions were estimated from RELION FSC curves with the 0.143 cutoff criterion^57,58^ and local resolutions were computed with MonoRes^59^. Final volumes were post-processed with LocaldeBlur using MonoRes volume as input in the case of the gp8 Titan Krios and tail data sets^60^.

### X-ray data collection and crystallographic processing

Crystals of gp8 portal protein were obtained by hanging-drop vapor diffusion in a number of conditions, but only a few of them diffracted. Gp8-13mer_cryst_ crystals were grown at 8.5 mg/ml protein sample in 15 % tacsimate, 0.1 M HEPES pH 7.0, 12% (w/v) PEG 3350 at 20 °C. Gp8_closed_ crystals were grown at 4.4 mg/ml protein sample in 0.2 M CaCl_2_, 0.1 M sodium acetate pH 4.6, and 30% (w/v) PEG 400 at 20 °C. All crystals were mounted in loops and flash frozen in liquid nitrogen, using a cryoprotective buffer. X-ray diffraction data were collected at ID29 and ID14-1 beamlines at the European Synchrotron Radiation Facility in Grenoble (France). Data were collected at 1.0679 Å and 0.9340 Å, and processed using XDS^61^.

A partial model (36% of the structure) of the gp8-13mer was built ab initio, using a 5.8 Å resolution cryo-EM map, and later used to obtain the initial crystallographic phases for the gp8-13mer crystallographic data by molecular replacement and phase extension. A monomer from the structure was used to phase the gp8_closed_ crystallographic data and as a template to trace the cryo-EM models of gp8_open_ and gp8 within the tail complex. Gp11 was traced on the cryo-EM tail map using T7 from gp11 protein threading model^27^ and TTPA crystal structure^33^ from KP32 as guides. Gp12 tail protein was traced ab initio using PSIPRED^62^ secondary structure prediction as a guide. All molecular replacement procedures were performed with PHASER^63^, and both cryo-EM and crystallographic models were traced in Coot^64^. During crystallographic model building, it was crucial to calculate density-modified maps, taking into account the presence of non-crystallographic symmetry^65^. PHENIX real-space refinement^66^ and REFMAC5^67^ were used to refine all the models, the latter within the CCP-EM suite for cryo-EM data^68,69^. All the models were validated using MolProbity^70^. Figures were prepared with Chimera^71^. In all electrostatic potential surfaces, blue represents 10 kcal/(mol e^−^) positive potential, whereas red represents −10 kcal/(mol e^−^) negative potential. Movies were prepared with the morphing option, interpolating the movement between two given conformations of the same protein with Chimera^71^.

### Reporting summary

Further information on research design is available in the Nature Research Reporting Summary linked to this article.

## Supplementary information

Supplementary Information

Reporting Summary

Description of Additional Supplementary Files

Supplementary Movie 1

Supplementary Movie 2

## Data Availability

The electron microscopy maps were deposited in the Electron Microscopy Data Bank (EMDB) with accession codes EMD-4667, EMD-4669, and EMD-4706. Atomic coordinates and crystallographic structure factors were deposited in the Protein Data Bank (PDB) under accession codes 6QWP, 6QX5, 6QXM, and PDB 6R21. All relevant data are available from the authors upon request.
