# Supplementary material for: Structures of T7 bacteriophage portal and tail suggest a viral DNA retention and ejection mechanism
Source: Nat Commun. 2019 Aug 20;10:3746. doi: 10.1038/s41467-019-11705-9 (PMC6702177; doi:10.1038/s41467-019-11705-9)
Supplement: Supplementary file 1 — Supplementary Information [file 41467_2019_11705_MOESM1_ESM.pdf]

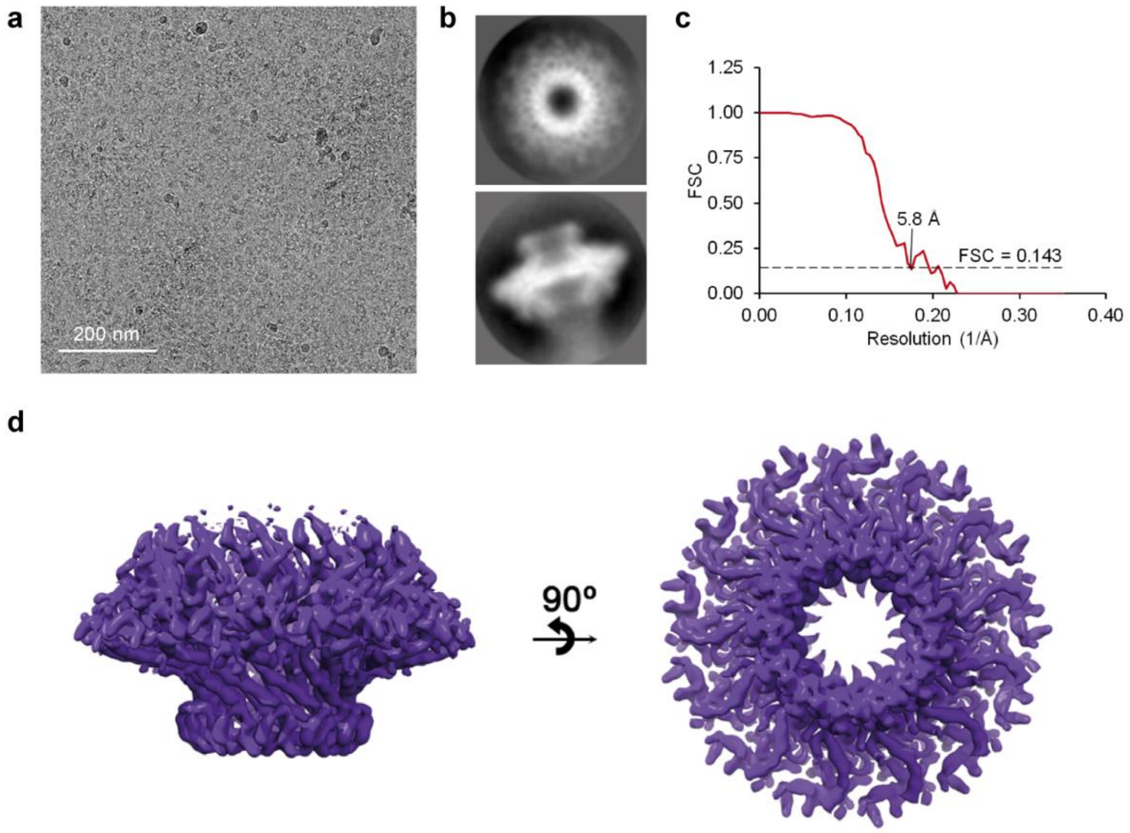

Supplementary Figure 1

Cryo-EM analysis of the gp8-13mer$_{EM}$ portal

a, Representative cryo-EM micrograph of the portal gp8 oligomer collected on a Talos Arctica 200 kV instrument with a Falcon II camera. The scale bar represents 200 nm. **b,** RELION two-dimensional (2D) averages showing end-on and side views of the oligomer (top and bottom images, respectively). **c,** Fourier shell correlation (FSC) curve. The FSC= 0.143 gold-standard cut-off criterion was used to determine the final resolution of the model (5.8 Å). **d**, RELION three-dimensional (3D) reconstruction showing a side view (**left**) and end-on view (**right**).

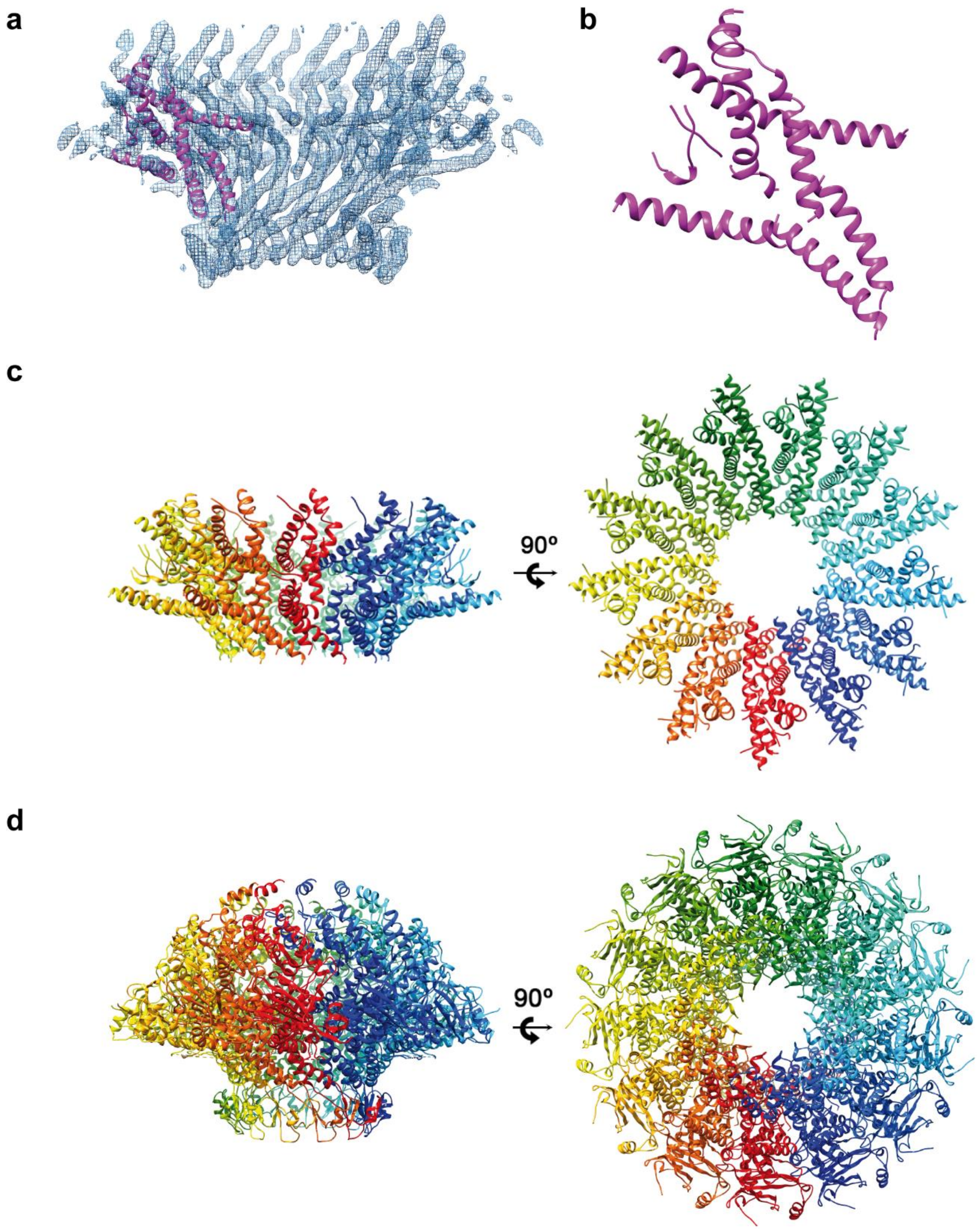

Supplementary Figure 2

Gp8-13mer structure solution, combining cryo-EM and X-ray crystallography

a, Cryo-EM density map used for model building. A partial model of a single subunit is fitted in ribbon representation. **b,** Ribbon representation of the single subunit partial gp8-13mer$_{EM}$ model, mainly composed of α-helices. **c,** Ribbon representation of side (**left**)

and end-on (**right**) views of the tridecameric initial model gp8-13mer$_{EM}$ generated from a cryo-EM density map, which was used for performing crystallographic molecular replacement against the gp8-13mer$_{cryst}$ X-ray data. Subunits are shown in rainbow coloring. **d,** Ribbon representation of side (**left**) and end-on (**right**) views of the gp8-13mer$_{cryst}$ complete refined model. Subunits were shown in rainbow coloring.

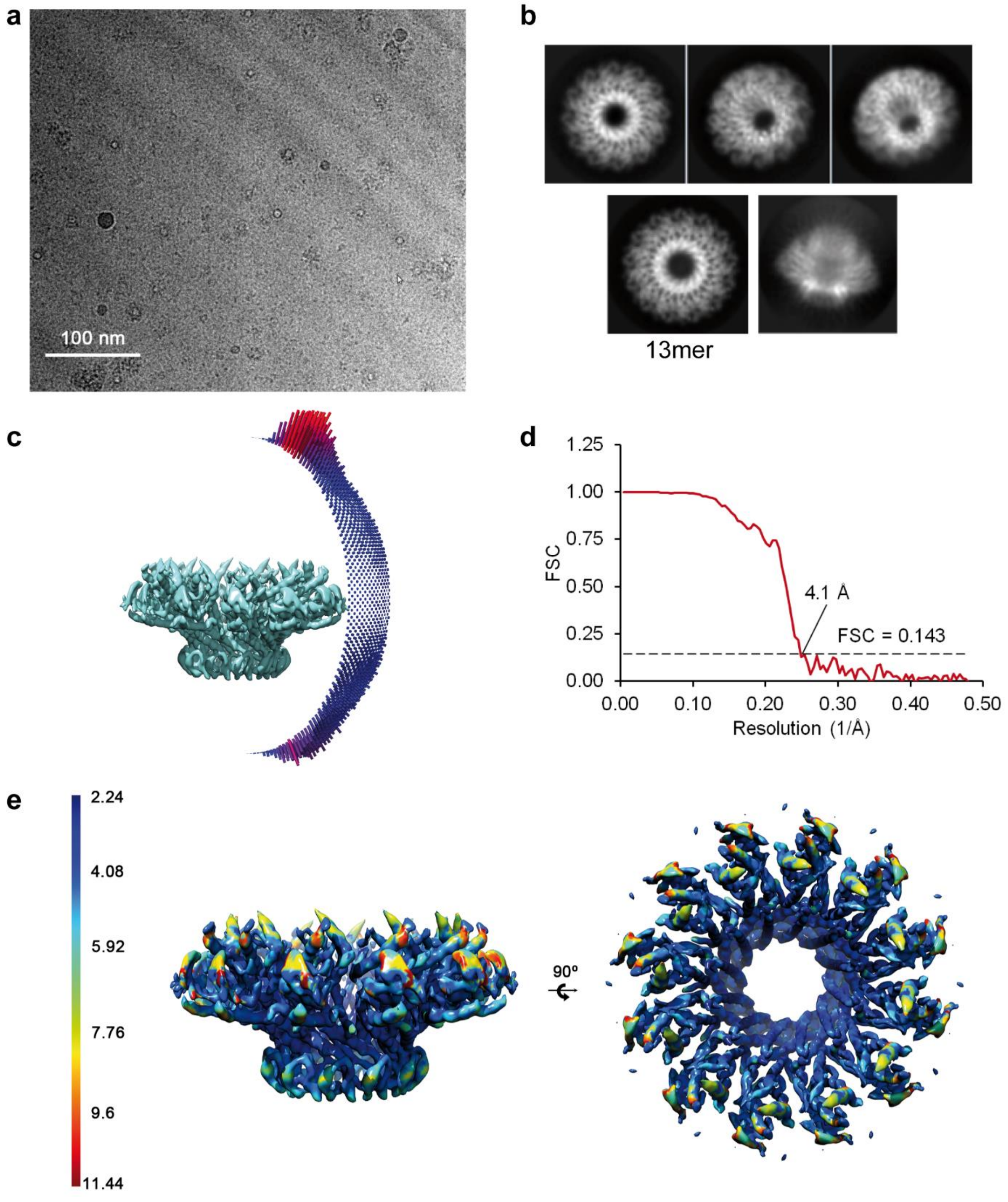
a
100 nm
b
13mer
c
d
1.25
1.00
0.75
0.50
0.25
0.00
FSC
4.1 Å
FSC = 0.143
0.00 0.10 0.20 0.30 0.40 0.50
Resolution (1/Å)
e
2.24
4.08
5.92
7.76
9.6
11.44
90º

Supplementary Figure 3

Cryo-EM analysis of the $gp8_{open}$ portal

a, Representative cryo-EM micrograph of the gp8 sample collected on a Titan Krios 300 kV instrument with a Gatan K2 camera. The scale bar represents 100 nm. **b,** RELION 2D averages showing end-on and side-views. All 2D averages correspond to 12mer particles, except for the bottom left average, which corresponds to 13mer particles. **c,** RELION 3D reconstruction of the model showing the angular distribution plot of the particles. **d,** RELION resolution FSC curve. The FSC= 0.143 gold-standard cut-off criterion was used to determine the final resolution of the model (4.1 Å). **e,** MonoRes local resolution map showing a side view (**left**) and an end-on view (**right**). The resolution plot ranges from 3.24-11.44 Å.

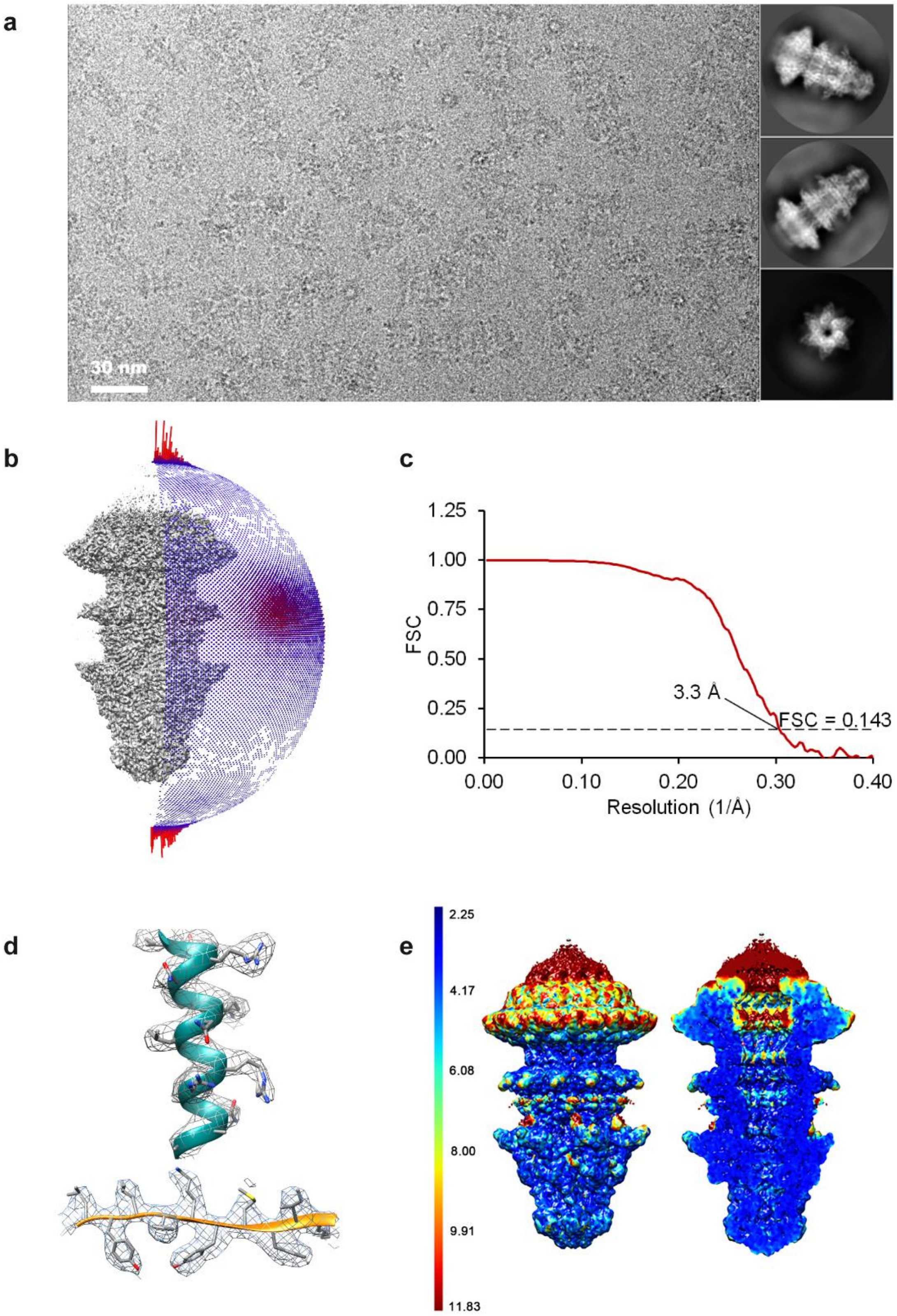
a
30 nm
b
c
1.25
1.00
0.75
0.50
0.25
0.00
FSC
3.3 Å
FSC = 0.143
0.00
0.10
0.20
0.30
0.40
Resolution (1/Å)
d
e
2.25
4.17
6.08
8.00
9.91
11.83

Supplementary Figure 4

Cryo-EM analysis of the gp8-gp11-gp12 tail complex

a, Left, representative cryo-EM micrograph showing gp8-gp11-gp12 tail complexes. The scale bar represents 30 nm. **Right**, RELION 2D averages. **b,** RELION 3D reconstruction of gp8-gp11-gp12 complex showing the angular distribution of the particles. **c,** RELION resolution FSC curve. The FSC= 0.143 gold-standard cut-off criterion was used to determine the final resolution of the model (3.3 Å). **d,** Extraction of the density from the 3D reconstruction corresponding to a α-helix and a β-sheet showing the placement of the side chains. **e,** MonoRes local resolution map, showing a side view (**left**) and a longitudinal cut (**right**). The resolution plot ranges from 2.25-11.83 Å.

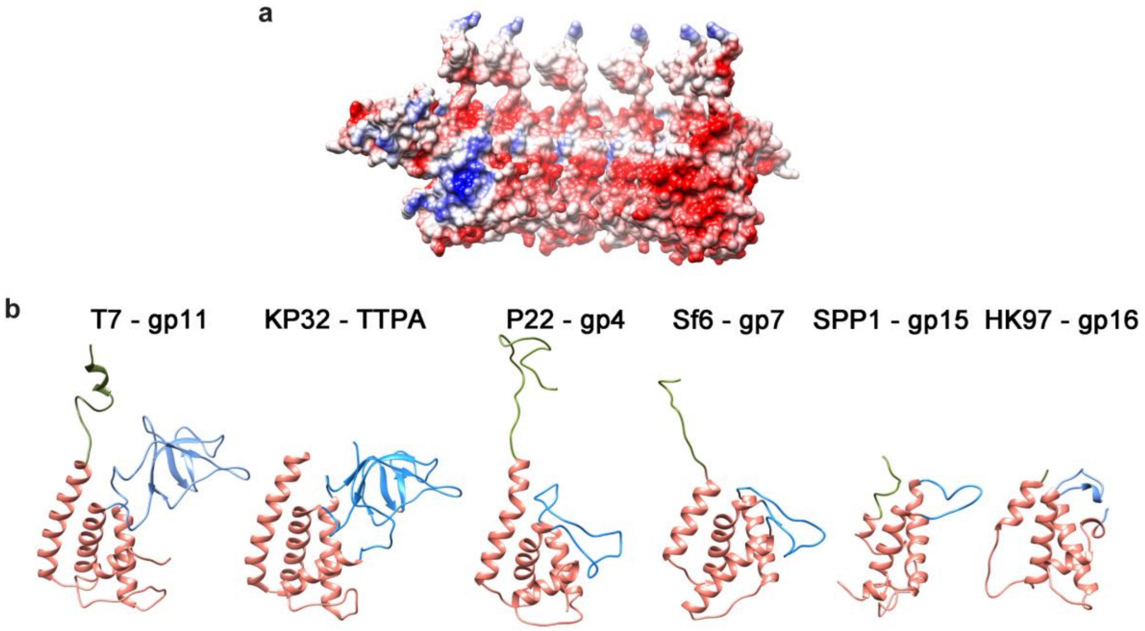

Supplementary Figure 5

Gp11 adaptor protein

a, Electrostatic potential surface of gp11 adaptor. **b,** Structural representations of T7, KP32 (PDB 5MU4), P22 (PDB 4V4K), Sf6 (PDB 5VGT), SSP1 (PDB 2KBZ) and HK97 (PDB 3JVO) adaptor proteins. The domains were colored as follows: embracing helix, light green; α-helix bundle, salmon and fiber dock, blue.

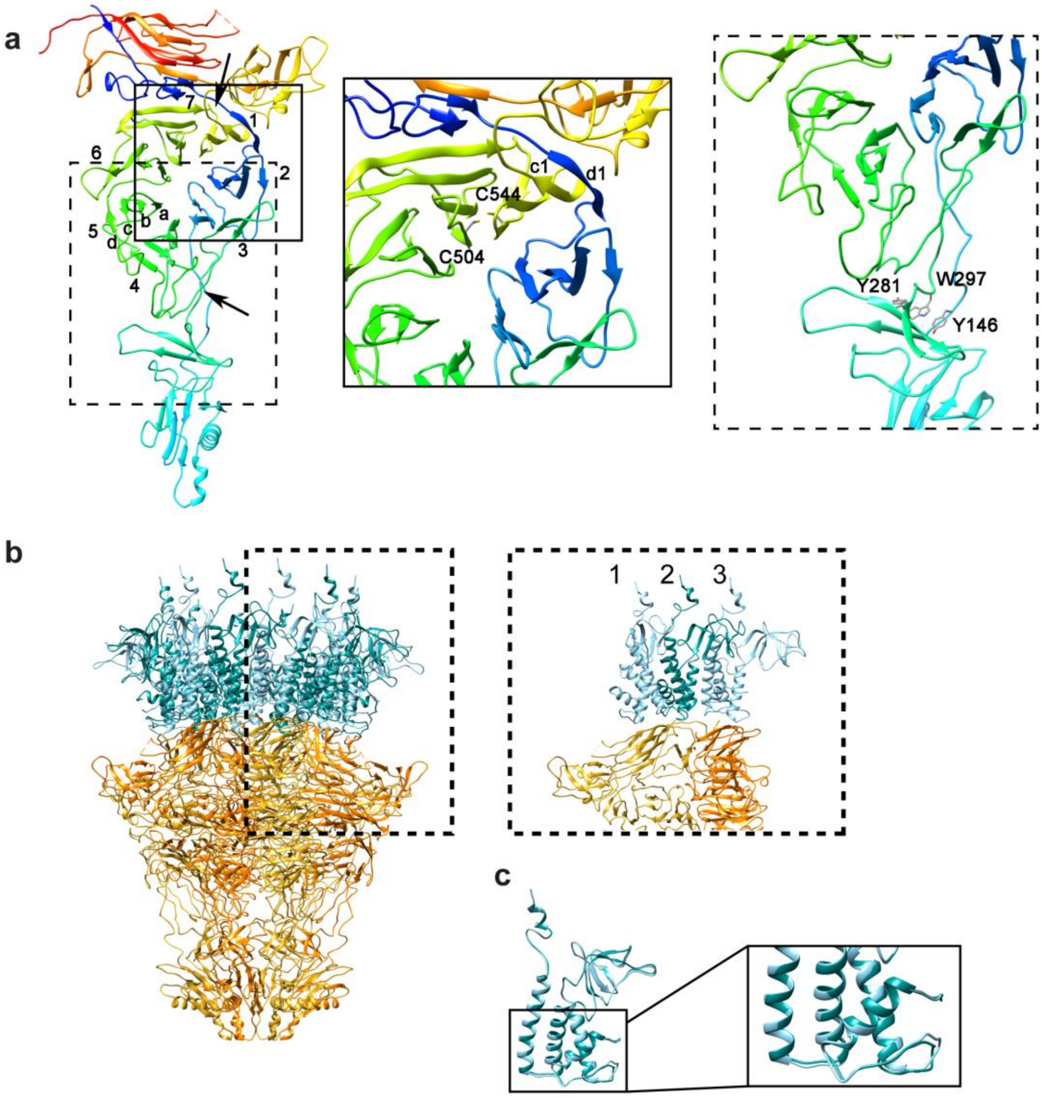

Supplementary Figure 6

Gp11 adaptor and gp12 nozzle tail proteins

a, Left, ribbon representation of gp12 protein colored from N-terminal (blue) to C-terminal (red). The 7 blades are numbered, and the order of the β-strands from a to d is indicated. Two arrows point to the two openings of the β-propeller. **Right**, Close-up view of the squares indicated in the left panel, where the residues involved in propeller stabilization are shown. **b, Left**, ribbon representation of gp11 adaptor and gp12 nozzle proteins in the tail complex. Gp11 monomers are shown in light blue and dark green and gp12 monomers in orange and yellow. **Right**, zoom of the gp11-gp12 interaction region. **c,** Comparison of gp11 monomers 1 and 2. **Right**, Close-up view of the loop region.

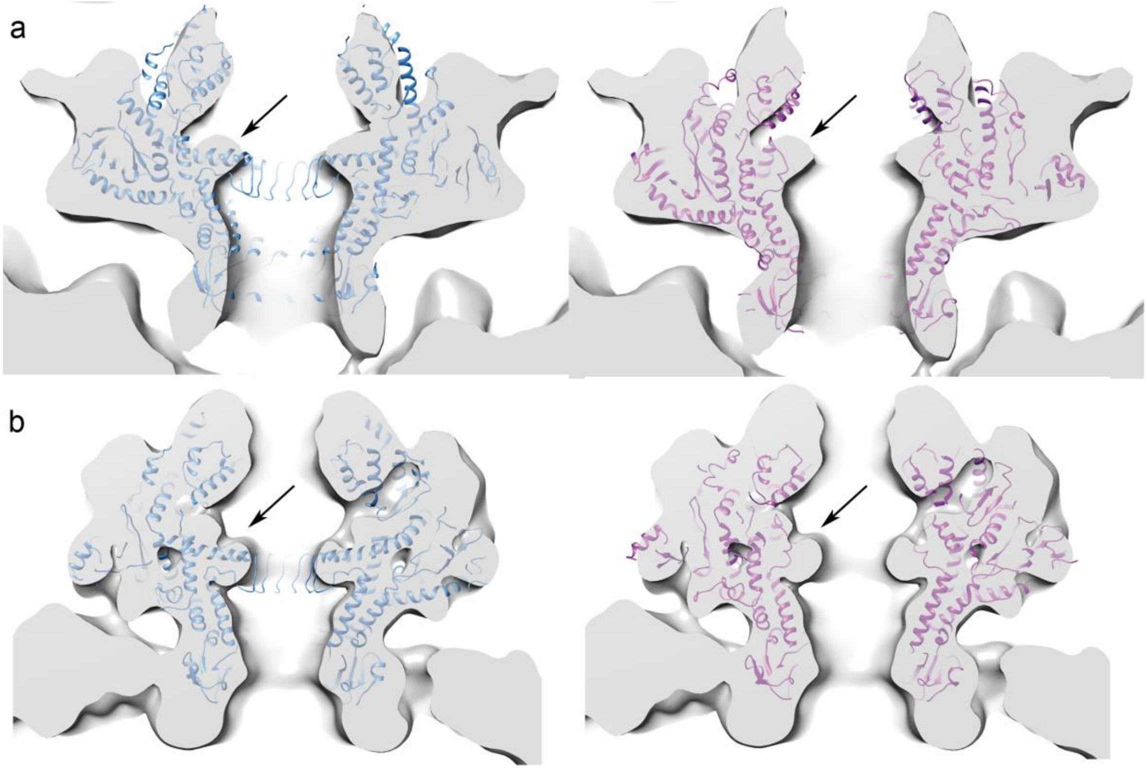

Supplementary Figure 7

Docking of the closed and open portal atomic structures into T7 and P23-45 proheads cryo-EM maps

a, Docking of T7 portal closed (**left**) and open (**right**) into the T7 prohead (EMD-1161, 24 Å resolution). **b,** Docking of T7 portal closed (**left**) and open (**right**) into the P23-45 prohead (EMD-4445, 9.33 Å resolution). The density corresponding to the tunnel valve of the portal region is shown with an arrow. This region is well filled with the atomic structure of the T7 portal when it is in its closed conformation (**left panels**), but not when it is open (**right panels**)

Supplementary Table 1: Cryo-EM data collection, refinement and validation statistics

	**gp8-13mer$_{EM}$ (EMD-4667)**	**gp8$_{open}$ (EMD-4669, PDB 6QXM)**	**gp8-gp11-gp12 (EMD-4706, PDB 6R21)**
**Data collection and processing**			
Nominal magnification	73,000	130,000	130,000
Voltage (kV)	200	300	300
Electron exposure ($e^-/Å^2$)	22.8	39.4	33.6
Defocus range (µm)	-1.0 to -3.0	-1.0 to -2.5	-1.0 to -3
Pixel size (Å/pix)	1.37	1.04	1.048
Symmetry imposed	C13	C12	C6
Initial particle images (no.)	180,911	193,945	213,367
Final particle images (no.)	12,642	32,688	92,382
Map resolution (Å)<br>FSC threshold	5.8<br>0.143	4.1<br>0.143	3.3<br>0.143
Map resolution range	3.78 to 12.18 Å	2.24 to 11.44 Å	2.6 to 11 Å
**Refinement**			
Initial model used	None (*ab-initio*)	gp8-12mer$_{cryst}$ / gp8-13mer$_{cryst}$	gp8-13mer$_{cryst,}$ gp11-threading and KP32-TTPA, gp12 none (*ab-initio*)
Map sharpening *B* factor ($Å^2$)	n.a.	n.a.*	n.a.*
Model composition<br>Non-hydrogen atoms<br>Protein residues	 n.a.<br>n.a.	 37,164<br>4,704	 100,992<br>12,786
B factors ($Å^2$)<br>Protein	 n.a.	 72.47	 93.60
R.m.s. deviations<br>Bond lengths (Å)<br>Bond angles (º)	 n.a.<br>n.a.	 0.008<br>1.129	 0.028<br>2.37
Validation<br>MolProbity score<br>Clashscore<br>Poor rotamers (%)	 n.a.<br>n.a.<br>n.a.	 2.61<br>6.67<br>8.63	 2.79<br>18.32<br>3.33
Ramachandran plot<br>Favored (%)<br>Allowed (%)<br>Disallowed (%)	 n.a.<br>n.a. 	 90.72<br>9.28<br>0.00	 83.63<br>15.68<br>0.69

n.a. not applicable, gp8-13merEM structure was not fully built and refined, only used for initial phasing

n.a.* not applicable, LocaldeBlur uses local resolution to apply a zone-depending sharpening

Supplementary Table 2: X-ray data collection and refinement statistics

	**gp8-13mer$_{cryst}$ (PDB 6QWP)**	**gp8$_{closed}$ (PDB 6QX5)**
**Data collection**		
Space group	$P4_22_12$	$P2_12_12_1$
Cell dimensions		
a, b, c (Å)	261.57, 261.57, 256.07	165.85, 191.33, 260.67
α, β, γ (º)	90, 90, 90	90, 90, 90
Resolution (Å)	3.40 (3.52-3.40)	3.60 (3.73-3.60)
R_{merge}	0.25 (1.19)	0.11 (1.08)
$I/\sigma(I)$	5.9 (1.3)	8.9 (1.1)
$CC_{1/2}$	0.969 (0.433)	0.997 (0.338)
Completeness (%)	95.70 (97.20)	89.70 (62.89)
Redundancy	3.4 (3.4)	3.6 (2.3)
**Refinement**		
Resolution (Å)	58.56 – 3.40	49.23 – 3.60
No. reflections	110,605	82,010
R_{work} / R_{free}	0.230 / 0.275	0.284 / 0.334
No. atoms		
Protein	49,530	45,048
B factors		
Protein	81.82	181.06
R.m.s. deviations		
Bond lengths (Å)	0.007	0.006
Bond angles (º)	1.2	1.45
Validation		
MolProbity score	2.27	3.17
Clashscore	6.01	21.96
Poor rotamers (%)	3.81	8.25
Ramachandran plot		
Favored (%)	91.61	86.02
Allowed (%)	7.27	11.45
Disallowed (%)	1.12	2.53

A single crystal was used for each structure. Values in parentheses are for highest-resolution shell.
