# Supplementary material for: Structures of T7 bacteriophage portal and tail suggest a viral DNA retention and ejection mechanism
Source: Nat Commun. 2019 Aug 20;10:3746. doi: 10.1038/s41467-019-11705-9 (PMC6702177; doi:10.1038/s41467-019-11705-9)
Supplement: Supplementary file 3 — Description of Additional Supplementary Files [file 41467_2019_11705_MOESM3_ESM.docx]

**Description of Additional Supplementary Files**

File Name: Supplementary Movie 1
Description: Portal channel valve movement (monomer). Morphing movie showing the conformational change in the gp8 portal channel valve monomer from its closed (as in the free portal) to its open conformation (as in the tail complex).

File Name: Supplementary Movie 2
Description: Portal channel valve movement (dodecamer). Morphing movie showing the conformational change in the gp8 portal channel valve from its closed (as in the free portal) to its open conformation (as in the tail complex).
